# Noble Gas Isotopes and Nitrogen Isotopologues Reveal Deep Sources and Subsurface Fractionation in Yellowstone Gases

**DOI:** 10.1021/acsearthspacechem.4c00349

**Published:** 2025-03-24

**Authors:** Michael W. Broadley, Peter H. Barry, Rebecca L. Tyne, David V. Bekaert, Ruta Karolyte, Michael R. Hudak, Katelyn McPaul, Carlos J. Ramirez, J. Curtice, Karen G. Lloyd, Christopher J. Ballentine, Bernard Marty, Edward D. Young, Alan M. Seltzer

**Affiliations:** 1 Department of Earth and Environmental Science, 5292 University of Manchester, Manchester M13 9PL, U.K.; 2 Marine Chemistry and Geochemistry, 10627 Woods Hole Oceanographic Institution, Woods Hole 02543, United States; 3 Universite de Lorraine, CNRS, CRPG, Nancy 54500, France; 4 Department of Earth Science, 6396 University of Oxford, Oxford OX1 3AN, U.K.; 5 Geosciences Department, 8609 Williams College, Williamstown 01267, United States; 6 Servicio Geológico Ambiental (SeGeoAm) Heredia, Heredia 40101, Costa Rica; 7 University of Southern California, Los Angeles, California 90007, United States; 8 Earth, Planetary and Space Science, UCLA, Los Angeles 90095, United States

**Keywords:** nitrogen, noble gases, mantle geochemistry, volatile accretion, mantle plumes, Yellowstone

## Abstract

Nitrogen plays a critical role in maintaining Earth’s hospitable surface environment over geological time. Despite our atmosphere being dominated by nitrogen, our understanding of how nitrogen was delivered to Earth and how subsequent planetary processes modified Earth’s nitrogen budget through time is currently lacking. Here, we report measurements of isotopologues of N_2_ (Δ_30_), along with ultrahigh precision measurements of Ar, Kr, and Xe isotopes, of hydrothermal gas samples from Yellowstone National Park. We show that δ^15^N variations are correlated with nonradiogenic Ar, Kr, and Xe isotope ratios, indicating that groundwater-derived nitrogen and noble gases in hydrothermal samples are fractionated by the same process as they diffuse through a rising column of magmatic CO_2_. Notably, a similar correlation exists regardless of the degree of atmospheric contamination, suggesting that the δ^15^N of the Yellowstone mantle source is similar to the atmosphere (i.e., ∼0‰). Two component mixing models between Δ_30_ and noble gases demonstrate that N_2_/^36^Ar (5.3 ± 0.7 × 10^5^) and ^36^Ar/^130^Xe (1611 ± 212) in the Yellowstone mantle source are lower and greater than the MORB mantle source, respectively, suggesting that contrary to previous findings, the plume mantle source has not been more efficiently overprinted by the addition of N_2_- and Xe-rich recycled material. Conversely, we suggest that the similarity in δ^15^N and N_2_/^36^Ar between the Yellowstone mantle source and chondritic meteorites indicates that nitrogen and noble gases in the deep mantle reflect the composition of the material that initially formed Earth.

## Graphical abstract

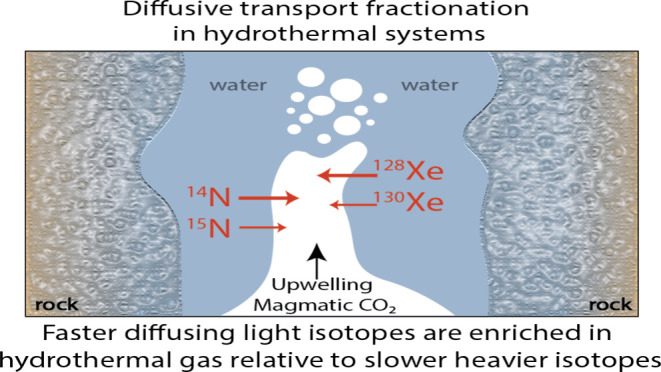

## 1 Introduction

Molecular nitrogen (N_2_) is the main constituent of Earth’s atmosphere and is an essential volatile element for life on Earth. Earth’s global nitrogen cycle is driven by plate tectonics, whereby nitrogen is emitted from Earth’s mantle through degassing at midocean ridges and mantle plumes, and returned to the mantle through the subduction of oceanic crust and sediments. Under present mantle oxidation conditions, N behaves as an incompatible element and is efficiently degassed to the atmosphere,^1^ with little isotopic fractionation.^2–4^ Subducted materials (i.e., oceanic crust and sediments) are enriched in ^15^N relative to atmosphere^5,6^ (positive δ^15^N, where δ^15^N = [(^15^N/^14^N)_sample_/(^15^N/^14^N)_air_–1]×1000). Subduction could therefore modify the nitrogen composition of the mantle over the billions of years since plate tectonic processes initiated.^7,8^ Furthermore, early differentiation processes such as magma ocean degassing^4,9^ and core formation^10–13^ may have also modified the nitrogen budget and isotopic composition of the Bulk Silicate Earth (BSE). Determining how these different planetary processes have modified Earth’s original N budget necessitates that the N isotopic composition of different mantle reservoirs is accurately determined.

Measured N-isotopes in midocean ridge basalts (MORB) and ocean island basalts (OIB), which originate from the convecting upper mantle and deep plume source mantle respectively, show generally limited variations, broadly ranging from δ^15^N of – 5‰ to +5‰.^7,14–17^ However, there is a consistent difference in δ^15^N between the MORB and OIB samples, with the δ^15^N of OIB samples (+3 ± 2‰;^7^) being consistently heavier than that measured in MORBs (−5 ± 3‰;^15–17^ ). The origin of this dichotomy between the two major geochemical mantle reservoirs is not clear, however the similarities in δ^15^N between OIB samples and sediments has been used to suggest N in the deep plume mantle source has been more efficiently overprinted from the addition of recycled material than the convecting MORB mantle reservoir.^7,8,18^ This assumes that both reservoirs inherited N from the same accretionary reservoir with low δ^15^N values, potentially from enstatite-like chondrite material (−20 ± 11‰^19^). However, determining whether different mantle reservoirs have preserved primordial nitrogen isotopic compositions is challenging due to the potential for N isotopes to be fractioned by planetary processes.

Combining N and noble gas isotopes can provide additional insights into the origin and evolutionary history of N in the mantle.^7,15^ This is because noble gases have several primordial isotopes (e.g., ^3^He, ^36^Ar, ^130^Xe), which can effectively trace their origin.^20,21^ In addition, because noble gases are chemically inert, they are unlikely to be modified by mantle differentiation processes that could modify the primordial N composition. Furthermore, heavy noble gases (Ar, Kr and Xe) are also efficiently recycled to the mantle, resulting in primordial compositions being largely overprinted by atmospheric signals.^22,23^ A coupled N and noble gas isotope approach could therefore identify primordial and recycled volatile signals in different mantle reservoirs.

To accurately determine primordial nitrogen and noble gas signatures in the mantle requires that samples be measured precisely enough to overcome ubiquitous air contamination and the large amount of recycled nitrogen and noble gases already present in the mantle. While air contamination is generally greater within hydrothermal gas samples than within solid basaltic samples, the essentially limitless quantity of hydrothermal gas available for analysis ensures that small isotope anomalies from air can be precisely measured. However, it has recently been shown that subsurface fractionation of groundwater-derived N_2_ ^24^ and noble gases^25^ in hydrothermal systems can generate light isotope enrichments, potentially masking the addition of air to these samples and mimicking primordial isotope signatures. The extent of isotopic fractionation measured within hydrothermal gas samples exceeds that expected for kinetic fractionation due to diffusion in water, for example during open system degassing.^24–29^ This suggests that boiling and phase separation within the hydrothermal system, while potentially able to account for the elemental fractionation of noble gases^30–32^ and nitrogen, is unlikely to account for the large extent of noble gas and nitrogen isotope fractionation measured within hydrothermal gas samples.^24,25^ The kinetic mass-dependent isotope fractionation was therefore previously ascribed to degassing of N_2_ and noble gases from groundwater at high temperature and pressure conditions, whereby gas solubilities could deviate considerably from behavior governed by Henry’s Law.^24,33^ However, more recent ultrahigh precision noble gas data from hydrothermal gas samples instead suggests that the degree of light isotope enrichment is consistent with diffusive transport fractionation (DTF),^25^ where atmospheric noble gases from groundwater are fractionated as they diffuse against a rising column of magmatic CO_2_. Therefore, it remains unclear whether the fractionation of groundwater-derived N_2_ and noble gases in hydrothermal gas samples are controlled by one or more physical processes.

In this study, we adopt a unique approach to identify fractionation processes that potentially mask mantle-derived N and noble gas isotope signatures. Only by accounting for any fractionation can we identify the composition of N and noble gases in the mantle. We couple the newly developed ^15^N^15^N tracer for atmospheric contamination^24^ with ultrahigh precision measurements of Ar, Kr and Xe isotopes by dynamic mass spectrometry,^25,34^ on gases collected within Yellowstone National Park. The measurement of ^15^N^15^N allows for the contribution of air contamination relative to a high temperature N_2_ source to be calculated based on the fact that atmosphere exhibits an extreme enrichment in ^15^N^15^N relative to N_2_ formed at high temperatures in thermodynamic equilibrium.^35,36^ The gas and thermal waters of Yellowstone have been extensively studied in the past and have shown evidence of subsurface fractionation due to boiling and phase separation.^30,37,38^ There is also clear evidence of mixing between different sources including air saturated groundwater, crustal and mantle-derived noble gases,^21,25,30,39,40^ which makes Yellowstone the perfect natural laboratory to study potential fractionation processes occurring within hydrothermal systems and better determine the volatile composition of the deep plume mantle endmember.

## 2 Materials and Methods

### 2.1 Gas Sampling

Samples were collected from several (n = 9) bubbling mudpots (Mud Volcano, Crater Hills and Obsidian Pool) as well as bubbling hot (Frying Pan) and cold (Brimstone Basin) hydrothermal springs, within Yellowstone National Park. At each degassing site, gas samples were collected by submerging a funnel into the bubbling mudpot or spring. Gases were flushed through silicone tubing, which was split into two streams to allow the simultaneous collection of gas into two large (1.5 L) Giggenbach bottles (pre-evacuated glass flasks containing 5N NaOH solution). This sampling method efficiently traps CO_2_, the major gas species in volcanic and hydrothermal emissions, therefore permitting large quantities of the nonreactive (i.e., noble gases and nitrogen) gases to be concentrated in the pre-evacuated headspace volume.^21,41,42^ In addition, 3 smaller (∼200 cm^3^) Giggenbach bottles were also collected for He isotopes and N_2_ isotopologue analysis.

### 2.2 Noble Gas Analysis by Dynamic Mass Spectrometry

Argon, Kr and Xe were analyzed by dynamic mass spectrometry, which yields a significant improvement in precision relative to traditional analyses by static noble gas mass spectrometer.^34^ To undertake the analysis of a given sample, the gas collected in the large 1.5 L Giggenbach bottle was expanded into a dedicated purification line, before being passed through a glass water trap immersed in a liquid N_2_-cooled ethanol slurry (−90 °C). The reactive gas species were then removed by exposure to a Ti-sponge getter held at 900 °C, before the heavy noble gases (Ar, Kr and Xe) were trapped onto a dip tube containing Si gel, immersed in liquid N_2_. The dip tube was then placed in a water bath held at 30 °C for 3 h to desorb the noble gases from Si gel before being connected to the dual inlet system of the isotope ratio mass spectrometer. For a more detailed outline of the purification and transfer protocol see Seltzer and Bekaert, (2022).^34^

The purified gas sample containing primarily Ar, Kr and Xe was measured using a Thermo 253 plus mass spectrometer equipped with 10 faraday collectors at Woods Hole Oceanographic Institute (WHOI). Pressure balancing between the sample and a reference gas with air-equilibrated water elemental ratios, was achieved by matching the intensities of the ^40^Ar beams in the sample and reference gas^34^. The isotopes of Ar are analyzed first before a magnetic peak jump is employed to measure the isotopes of Xe, followed by Kr. Each analysis consists of 48 cycles of Ar measurements, 96 cycles of Xe, and 64 cycles of Kr, with each block representing a sample gas measurement intermediate between two measurements of the reference gas. Small corrections are applied to correct for instrumental nonlinearity and matrix effects, as detailed in Seltzer and Bekaert, (2022).^34^

### 2.3 Helium Isotope Analysis by Static Noble Gas Mass Spectrometry

To limit He diffusion out of the glass Giggenbach bottles over time, several aliquots of gas were expanded and sealed into pre-evacuated copper tubes, within 6 weeks of being collected. The gases within these Cu tubes were then analyzed for their He isotopes (and nitrogen isotopologues; see section 2.4). For the analysis of He isotopes, Cu tube samples were connected to a dedicated extraction line, at WHOI (see Barry et al., 2022 for methods^43^), using an O-ring connection. In brief, an aliquot (∼5 cm^3^) of gas was expanded from the Cu tube into a dedicated extraction line where the pressure of the gas was monitored using a capacitance manometer. The gas was then purified (i.e., reactive gases were removed) by exposing them to a Ti sponge held at 650 °C. After 10 min, the temperature of the Ti sponge was reduced to room temperature in order to trap hydrogen. The remaining gas was further purified through exposure to one hot (250 °C) and one room temperature SAES ST707 getter. Helium was then separated from the other noble gases by trapping all the remaining gases on a stainless steel cryo trap held at 10 K. The cryotrap was subsequently raised to 30 K, therefore releasing the He for analysis on the Nu Noblesse mass spectrometer. Mass discrimination and reproducibility were monitored through the automated analysis of overnight air standards. Blanks were monitored weekly and were consistently less than 1% of the ^4^He signals.

### 2.4 Nitrogen Isotopologue Analysis

A subset of the samples (n = 8) were measured for their nitrogen isotopologue composition at UCLA. Molecular nitrogen (N_2_) was purified on a vacuum line interfaced with a gas chromatography (GC) system.^44^ Cu tubes containing gas extracted from small Giggenbach bottles were attached to the vacuum line using O-rings. The gas was then passed through a water trap held at – 20 °C using ethanol cooled by liquid N_2_. The gas was then drawn down onto a Si-gel trap on the preparation line at liquid N_2_ temperature. Gas was subsequently released from the trap by heating it with a heat-gun, while a helium carrier gas simultaneously flushed the gas through the GC column. As nitrogen exited the GC it was trapped on a separate liquid N_2_-cooled Si-gel trap. This allowed for the separation of N_2_ from the other major gas phases present in the sample (Ar, O_2_ and CH_4_). Helium was then pumped away, before the gas was released from the Si-gel trap at ∼ 60 °C. Finally, the gas was trapped on a Si-gel containing pyrex dip tube cooled by liquid N_2_. The dip tube of purified N_2_ gas was then transferred to the dual inlet system of the Nu Instruments Panorama mass spectrometer at UCLA. Prior to the start of the analysis, the sample was equilibrated over a period of 30 min within the bellows of the dual inlet system.

The ^14^N^14^N and ^14^N^15^N isotopologues were determined on the Panorama mass spectrometer using a 10^11^ Ω faraday cup, while ^15^N^15^N was measured using a secondary electron multiplier. The high mass resolving power of the Panorama permitted the interferences from ^14^N^16^O and ^12^C^18^O to be effectively resolved from the ^15^N^15^N peak.^44^ All samples were analyzed in 8 blocks over a period of 7.5 h,^24^ yielding an internal precision of 0.1‰ (1σ) on the Δ_30_ (Δ_30_ = ^30^R/(^15^R)^2^–1 (‰), where^30^R = ^15^N^15^N/^14^N^14^N and^15^R = ^15^N/^14^N). Aliquots (2 cm^3^) of air collected outside the Geology Building at UCLA, were run intermittently throughout the analytical campaign, yielding an average Δ^30^ of +18.9 ± 0.6‰ (2 s.d.), which is indistinguishable from previous determination^35^ of the atmospheric Δ^30^ value (+19.2 ± 0.3‰ (2 s.d.)). Due to the large amount of gas available for analysis, blank contributions were negligible.

## 3 Results

Noble gas isotope compositions are consistent with previous measurements of hydrothermal gas from within Yellowstone National Park.^21,25,40,45,46^ Measured ^3^He/^4^He values range from 3.0 ± 0.1 R_A_ in Brimstone Basin to 16.8 ± 0.7 R_A_ in Obsidian Pool. The high ^3^He/^4^He measured in Obsidian Pool, which sits near the center of the present-day caldera, likely originates from the underlying Yellowstone mantle plume, while the low ^3^He/^4^He in Brimstone Basin is consistent with a large contribution of radiogenic ^4^He from the underlying cratonic crust to the east of the Caldera.^40^ The large range in ^3^He/^4^He between the different sites in Yellowstone National Park is likely due to variable contributions of crustal-derived ^4^He to the upwelling primordial helium (i.e., high ^3^He/^4^He) from the Yellowstone mantle plume.^21,40^

Heavy noble gas (Ar, Kr and Xe) isotope compositions of the gas, as measured by dynamic mass spectrometry, are dominated by an atmosphere-like component. All Ar, Kr and Xe isotopes measured by dynamic mass spectrometry in this study are reported as per mil (‰) deviations relative to atmosphere using delta notation, where a given isotopic or elemental ratio (noted R) is expressed as δ*R*=(*R*_sample_/*R*_atmosphere_–1) × 1000. The δ^40^Ar/^36^Ar values of samples range from +19.17‰ ± 0.01‰ in Frying Pan Spring to +1010.85‰ ± 0.04‰ in Obsidian Pool. The range of δ^40^Ar/^36^Ar measured in Brimstone Basin (+42.75‰ ± 0.01‰ to +163.00‰ ± 0.01‰) measured in this study is significantly lower than the maximum values measured previously (>+3500‰),^21^ suggesting a far greater contribution from an atmosphere-derived component in the 2022 samples compared to those collected in 2018. Large variations in ^40^Ar/^36^Ar also exist across different degassing sites within single localities, e.g., the two samples collected at Obsidian Pool range from +347.37‰ ± 0.01‰ up to +1010.85‰ ± 0.04‰. Furthermore, samples collected simultaneously at the same degassing site can vary in δ^40^Ar/^36^Ar by ∼ 100‰, suggesting that gas compositions evolve over the few hours during which the Giggenbachs were being filled, highlighting the dynamic nature of hydrothermal systems.

The nonradiogenic isotopes of Ar (^36,38^Ar) and Xe (^128,130^Xe), as well as Kr isotopes minorly affected by fissiogenic decay of ^238^U (^82,84,86^Kr), show highly correlated enrichments in the light isotopes relative to atmosphere (Figure 1). The light isotope enrichment of Ar, Kr and Xe is consistent with previous high precision analyses of hydrothermal gas from Yellowstone and other volcanic regions, suggesting that there is a pervasive subsurface fractionation process occurring within hydrothermal systems.^25^ The maximum extent of fractionation measured within the Yellowstone samples is – 8.36‰ ± 0.01‰, – 3.01‰ ± 0.02‰ and +1.79‰ ± 0.15‰, for ^38^Ar/^36^Ar, ^86^Kr/^84^Kr (Figure 1a) and ^128^Xe/^130^Xe (Figure 1b), respectively. The majority of samples also exhibit excesses in ^129^Xe/^130^Xe and the fissiogenic isotopes ^131,132,134,136^Xe (Supplementary Table), which are greater than would be expected from any fractionation processes, but consistent with a contribution from mantle-derived Xe.^21^

**Figure 1:**
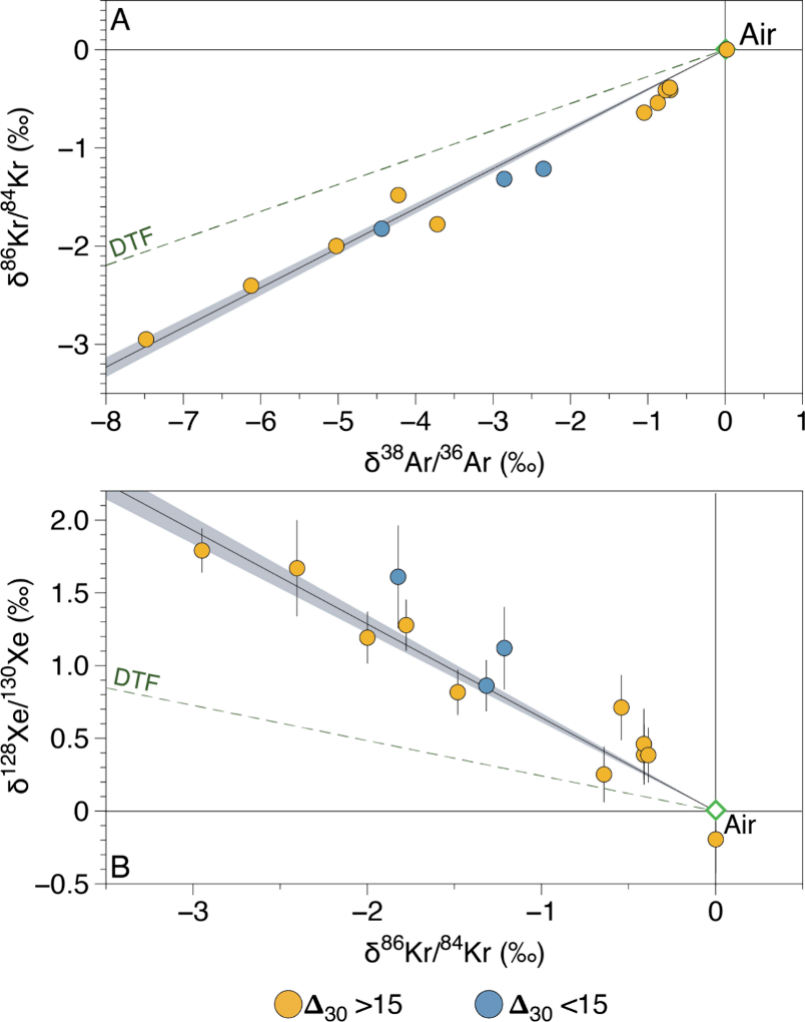
Stable Ar (A), Kr (A, B) and Xe (B) isotope variations measured in hydrothermal gas samples from within Yellowstone National Park. Samples are reported in delta notation relative to the atmosphere. Predictions for steady-state isotope fractionation by diffusive transport fractionation (DTF) of noble gases through CO_2_ is shown. Samples are color coded based on their Δ_30_ (see Figure 2). Linear regression represents an error weighted fit shown with 1σ uncertainty envelope. Uncertainties for the samples are reported to 1σ and are often smaller than symbol size.

Samples display consistently negative δ^15^N values with respect to atmosphere and range from – 3.83‰ to – 0.55‰ (Figure 2). This range is significantly lower than the conventional values assumed to represent nitrogen originating from the plume source mantle (∼+3‰).^48^ The Δ_30_ values range from 8.64 to 18.61 (Figure 2) and are consistent with a mixture of atmospheric (Δ_30_ = +19.2 ± 0.3‰) and high temperature N_2_ components in thermodynamic equilibrium (Δ_30_ ∼ 0‰). The range in δ^15^N and Δ_30_ measured in this study (Figure 2) are consistent with previous measurements of gas from Yellowstone.^24^ There is however no significant correlation between δ^15^N and Δ_30_ data, suggesting that variations in δ^15^N are not the result of simple two component mixing between an atmospheric component, with a single δ^15^N composition and a high temperature component (Figure 2).

**Figure 2:**
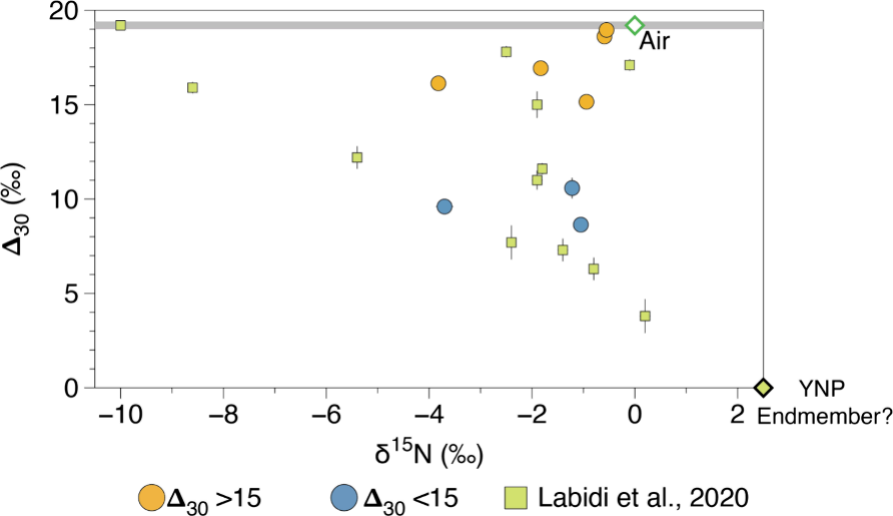
Nitrogen isotope and isotopologue composition of hydrothermal gas samples from Yellowstone. The nitrogen isotopic and isotopologue compositions of air are shown. The atmospheric composition of Δ_30_ is +19.1 ± 0.3‰, relative to high-temperature nitrogen with a Δ_30_ = 0‰.^35^ New δ^15^N data reported in this study range between – 4‰ and – 1‰ and show no significant correlation with Δ_30_. Previously analyzed samples from Yellowstone are also shown, as well as the previously suggested endmember value for the Yellowstone mantle plume.^24^ The lack of correlation between δ^15^N and Δ_30_ suggests there is not a single fractionated air component within the hydrothermal system, therefore extrapolating mixing lines though samples to an endmember composition^24^ is likely not justified.^47^ Samples with the lowest Δ_30_ cluster around – 1‰ for δ^15^N suggesting this is the best estimate for the Yellowstone mantle source. Uncertainties for the samples are reported to 1σ and are often smaller than symbol size.

## 4 Discussion

### 4.1 Consistent Subsurface Fraction of Nitrogen and Noble Gas Isotopes in Hydrothermal Gas

Yellowstone samples display similarly large (i.e., per mil level) anomalies in the stable Ar, Kr and Xe isotopes relative to atmosphere (Figure 1), as was previously identified in other hydrothermal gas samples worldwide.^25^ We find that all samples fall along a single trend which passes through the atmospheric composition (Figure 1). This is true regardless of whether the samples contain a high temperature N_2_ component (as defined by nonatmospheric Δ_30_ values) or not. The singular trend in these data, despite variations in the degree of high temperature mantle contributions between the samples, suggests that the nonradiogenic Ar, Kr, and Xe isotopes in Yellowstone National Park samples are dominated by an atmospheric component that has been variably affected by some secondary fractionation process­(es) and does not primarily reflect contributions of primordial noble gases from the mantle.^25,49^

Nonradiogenic isotope variations of Ar, Kr and Xe in Yellowstone gases are correlated with δ^15^N (Figure 3), suggesting that both nitrogen and noble gases within hydrothermal systems are fractionated by a similar process. This would appear to rule out a nitrogen specific fractionation processes such as the oxidation of crustal NH_3_, which can form isotopically light N_2_ ^51^ and potentially randomly distribute ^15^N and ^14^N atoms resulting in a Δ_30_ of 0‰.^52^ Furthermore, the strong correlation between Δ_30_,^40^Ar/^36^Ar and ^129^Xe/^130^Xe (Figure 4), which are sensitive tracers of mantle input, suggests that N_2_ within the Yellowstone hydrothermal system is best explained as a mixture between a deep mantle component and a variably fractionated groundwater-derived atmospheric component. Bekaert et al., (2023)^25^ previously demonstrated that the negative correlation between ^84^Kr/^36^Ar (or ^132^Xe/^36^Ar) and δ^15^N followed a similar trend to that predicted by diffusive transport fractionation (DTF), and did not require nonidealized degassing at extreme temperature and pressure as previously suggested.^24^ However, elemental noble gas ratios are not ideal for tracking fractionation processes as they show a wide range of compositions in hydrothermal systems due to differences in the equilibration temperature of the ASW component, as well as potential differences arising from water-rock interactions and high-temperature vapor–liquid partitioning.^32,53^ Since these processes do not induce significant isotopic fractionation, at least at the scale measured here, the strong correlation between δ^15^N and the stable noble gas isotopes (Figure 3A,B,C) suggests that processes that were shown to pervasively affect noble gases in the subsurface, similarly affect nitrogen isotopes.

**Figure 3:**
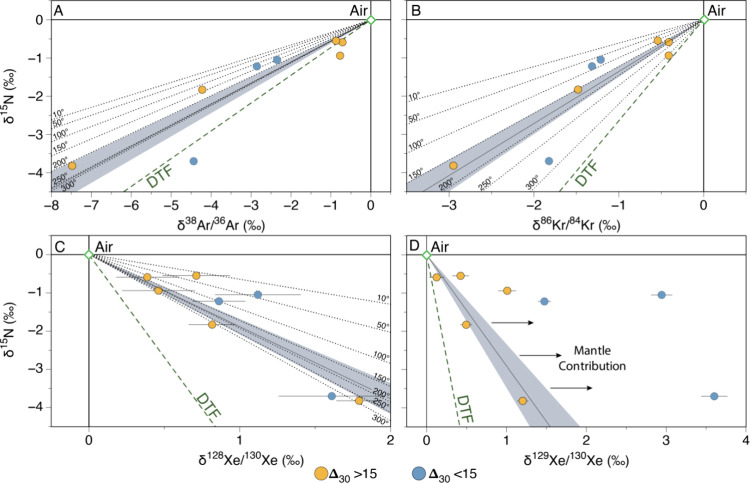
Ar (A), Kr (B), ^128^Xe (C) and ^129^Xe (D) isotope variations relative to δ^15^N in hydrothermal gas samples from within Yellowstone National Park. Samples are reported in delta notation relative to the atmosphere. Samples form a single correlation in panels A, B and C regardless of Δ_30_ suggesting the Yellowstone mantle source has a similar δ^15^N composition to the atmosphere value. There are however two distinct correlations when δ^15^N is plotted against δ^129^Xe/^130^Xe (Panel D). Samples with high Δ_30_ are offset from the trend formed by the low Δ_30_, likely due to the addition of high δ^129^Xe/^130^Xe from the mantle. The best fit linear correlations are slightly offset from the steady-state isotope fractionation by diffusive transport fractionation (DTF), with the difference between expectation and reality increasing from N_2_/Ar to N_2_/Xe. The black dotted lines represent DTF offset by the differences in solubility between N_2_ and stable Ar (A), Kr (B) and Xe (C) gas in waters at different temperatures ranging from 10 to 300 °C.^50^ Solubilities are not shown in (D) due to the variable input from mantle-derived ^129^Xe in the samples. There is a broad agreement between the error weighted fit of the data and the solubility ratios for nitrogen vs noble gases in water at temperatures greater than ∼ 200 °C. Linear regression represents an error weighted fit shown with 1σ uncertainty envelope. The linear regression for panel D uses only those samples with Δ_30_ > + 15, which are dominated by atmosphere. Uncertainties for the samples are reported to 1σ and are often smaller than symbol sizes.

**Figure 4:**
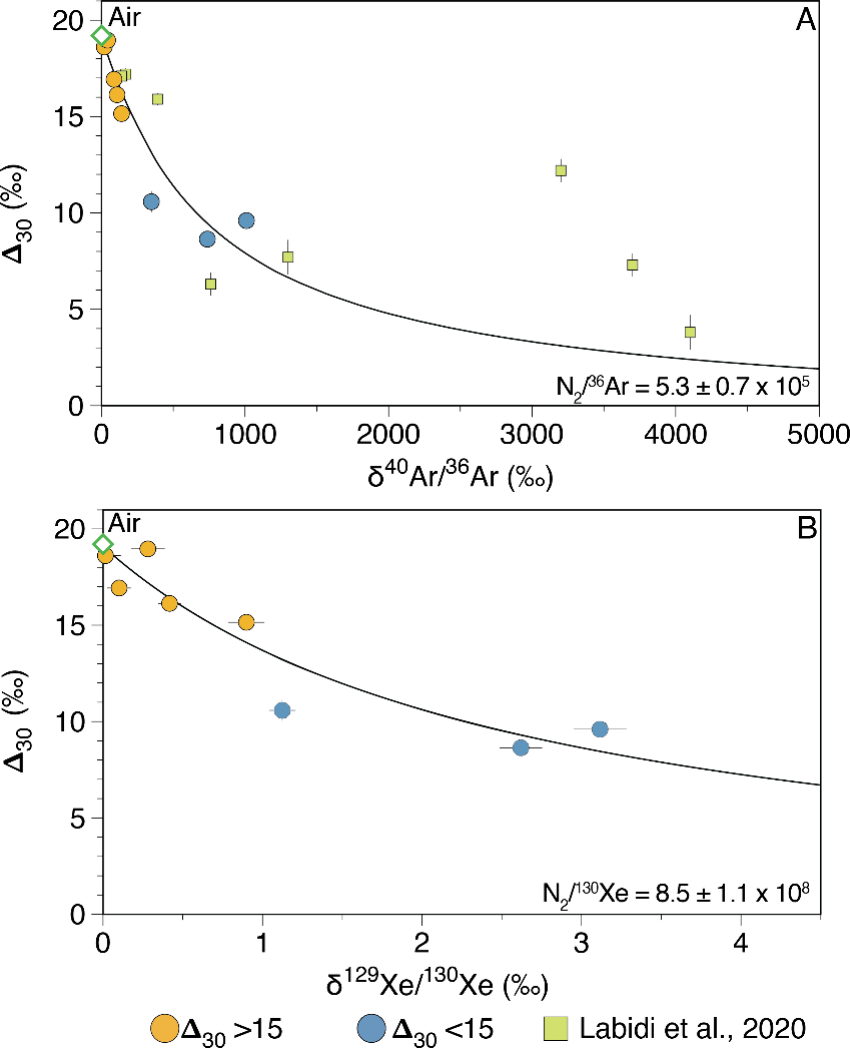
Relationship between Δ_30_ and δ^40^Ar/^36^Ar (A) and δ^129^Xe/^130^Xe (B) in hydrothermal gas samples from Yellowstone. The data form a strong correlation which represents two component mixing between atmosphere and a high temperature nitrogen component. The correlation between Δ_30_ and δ^129^Xe/^130^Xe confirms that the high temperature low Δ_30_ nitrogen component in the samples originates from the mantle. The fit of the mixing lines is calculated by assuming the Yellowstone mantle has a δ^40^Ar/^36^Ar, δ^129^Xe/^130^Xe and Δ_30_ of ∼ + 35000‰, + 76‰^19^ and 0‰ respectively. The curvature of the mixing lines yields a N_2_/^36^Ar and N_2_/^130^Ar of 5.3 ± 0.7 × 10^5^ and 8.5 ± 1.1 × 10^8^, respectively. Previously analyzed data from Yellowstone broadly fit the new data, however, the samples with high δ^40^Ar/^36^Ar likely contain a crustal component with high N_2_/^36^Ar, which causes them to fall off the mixing line. Uncertainties for the samples are reported to 1σ and are often smaller than symbol size.

Diffusive transport fractionation readily explains many of the geochemical features observed in hydrothermal gases worldwide, including the large degree of fractionation, the consistent enrichment in light isotopes, and the fact that no sample has yet exceeded the maximum degree of fractionation predicted by steady state DTF (i.e., around – 14‰ for δ^38^Ar/^36^Ar, – 4‰ for δ^86^Kr/^84^Kr, + 2‰ for δ^128^Xe/^130^Xe and – 10‰ for δ^15^N).^24,25^ When considering a single elemental species (e.g., Kr in Figure S1), the expected DTF fractionation line accurately predicts the range of values measured within hydrothermal gas samples. However, it is clear that our data do not perfectly fit the trajectory expected for DTF when looking at different elements together (Figure 1 and 3), suggesting that the kinetics of isotopic fractionation (i.e., how “quickly” isotope fractionation reaches steady state) may differ across elements. For example, when δ^15^N is plotted against δ^38^Ar/^36^Ar, δ^86^Kr/^84^Kr and δ^128^Xe/^130^Xe, the slopes of the best fit lines through the data are consistently less steep than the predicted trajectories for steady state DTF for all elements against a CO_2_ gas phase (Figure 3). The difference between the slopes predicted by steady state DTF for all elements and the measured trends is smallest for δ^15^N vs ^38^Ar/^36^Ar, and increases systematically when δ^15^N is plotted against Kr and Xe (Figure 3), respectively. For example, the maximum δ^128^Xe/^130^Xe measured from the Yellowstone samples (+1.79‰ ± 0.01‰) is close to the expected steady-state isotope fractionation expected for Xe diffusion against CO_2_ (+2‰), despite the δ^15^N (−3.82 ± 0.01‰) of this sample being significantly far away from the steady-state fractionation predicted for the DTF of N_2_ against CO_2_ (−10‰). This suggests that, while DTF is the dominant process controlling the isotope ratios of volatiles in hydrothermal gas species, there is another process involved which determines the ease with which an element reaches steady-state. This process appears to be mass dependent, with Xe being the closest to steady-state, followed by Kr, Ar and then N_2_.

The mass dependent nature of diffusion against CO_2_ (i.e., DTF) was previously described for Ar, Kr and Xe^25^. These authors suggested that the diffusion rate of the different noble gas elements out of a water phase into the CO_2_ gas phase (i.e., piston velocity ratio), had an influence on the degree of DTF experienced by different volatiles elements. For example, Ar being the fastest diffusing element would be more likely to reach isotopic equilibrium within the CO_2_ phase than heavier and slower diffusing Kr and Xe. Argon would therefore be less likely to be kinetically fractionated and reach steady state DTF than Kr and Xe. However, since Kr and N_2_ have similar diffusion coefficients in water,^54,55^ there should not be a significant offset of the data for Kr and N_2_ from the steady-state DTF trend, which is not the case (Figure 3B). Therefore, differences in diffusivity are unlikely to control the different degree of DTF experienced by N_2_ and the different noble gas elements.

An alternative possibility is that differences in solubility have a controlling influence on how readily an elemental species reaches equilibrium as it diffuses against CO_2_. To explore this possibility, we plot the solubility ratios for N_2_ vs Ar, Kr and Xe^50^ at different temperatures ranging from 10 to 300 °C (Figure 3). Despite some spread in the data, which is expected given that the samples come from a variety of sampling sites, there is broad agreement between the fit of the data and the solubility ratios for nitrogen vs noble gases in waters at temperatures greater than ∼ 200 °C. This suggests that, deep within the hydrothermal system, solubility-controlled partitioning of noble gases and nitrogen out of a water phase into the CO_2_ gas phase may control how readily a gas species reaches isotopic equilibrium with the CO_2_ phase. Because N_2_ is the least soluble in water, it is most likely to reach isotopic equilibrium with the CO_2_ phases, thereby limiting the degree of kinetic isotope fractionation.

The temperatures suggested by the data relative to the solubility ratios are consistent with temperature estimations of fluids from within the Yellowstone hydrothermal system (170 to 310 °C) based on CO_2_–CH_4_–CO-H_2_O–H_2_ gas equilibration reactions.^56^ We note however that the solubility coefficients shown for N_2_ vs Ar, Kr and Xe may not be appropriate when a dense CO_2_ phase is present.^33^ Instead, under such conditions, gas solubilities can deviate considerably from behavior governed by Henry’s Law, with the affinity for the CO_2_ phases increasing the most for Xe, followed by Kr, and then Ar (and then presumably N_2_, although to our knowledge no experimental data exist).^33^ In the case of high CO_2_ densities expected for a hydrothermal system, the role of molecular interactions between the noble gases and CO_2_ may play a role. For example, the greater polarizability (which increases with atomic size) of Xe relative to Kr, and then Ar, could increase the potential for molecular interactions with CO_2_ to occur, therefore impeding diffusion and limiting the potential for Xe to reach equilibrium in the gas phase, and making Xe more prone to kinetic fractionation. It is clear that further experimental work is needed to investigate the diffusive fractionation processes of gas species within CO_2_–H_2_O systems that would better replicate the conditions present within deep hydrothermal systems. However, regardless of the exact mechanism(s) which control(s) the degree to which each element reaches steady state fractionation, it appears clear that DTF of groundwater-derived gases against rising CO_2_ is the main cause of isotopic fractionation^25^ observed in both noble gases and nitrogen within hydrothermal gas samples.

### 4.2 The Nitrogen Isotopic Composition of the Yellowstone Mantle Source

Nitrogen isotopologues of hydrothermal gases provide a unique means to disentangle deep source nitrogen isotope signatures from fractionation brought about by diffusive transport fractionation (DTF; section 3.1). When comparing δ^15^N with stable noble gas signatures (Figure 3) there is no significant difference between samples that have low (<+15‰) and high (>+15‰) Δ_30_ (Figure 3). Yet, these two populations of data are thought to contain different contributions of deep (presumably mantle-derived), and atmosphere-derived nitrogen. This could suggest that either: (i) high and low temperature components both have similar N isotopic source signatures, or (ii) the majority of N_2_ in the samples originates from groundwater-derived atmosphere, similar to the stable noble gases. In the second case this would require that Δ_30_, which traces N_2_ at high temperature equilibrium, may not be tracing the input of mantle nitrogen but rather high temperature formation of N_2_ deep within the hydrothermal system.^57^ Previously, the negative correlation between Δ_30_ and δ^40^Ar/^36^Ar (Figure 4) was interpreted as Δ_30_ tracing the addition of mantle N_2_. However, since ^40^Ar is continually produced in both the crust and mantle from the decay of ^40^K, it remained somewhat ambiguous whether the nitrogen in samples with low Δ_30_ originated from the mantle or the crust.^24,57^ However, the strong negative correlation between Δ^30^ and δ^129^Xe/^130^Xe, where excess ^129^Xe is clearly associated with mantle inputs from the decay of extinct ^129^I (*T*_1/2_ = 16 Ma), confirms that Δ_30_ is an effective tracer of mantle nitrogen inputs within Yellowstone hydrothermal systems. This correlation therefore represents mixing between an atmospheric and a mantle component. The preservation of mantle-derived N isotopes and the radiogenic and fissiogenic isotopes of Xe in hydrothermal gas, indicates that mantle volatiles signatures can resist complete atmospheric overprinting. In the case of N isotopes, the larger concentration of N_2_ with magmatic gas, combined with the lower solubility of N_2_ in groundwater, when compared to the noble gases, may result in the preferential retention of mantle signatures relative to the stable nonradiogenic noble gas isotopes. The radiogenic and fissiogenic noble gas isotopes on the other hand often retain mantle-derived signatures, even when the stable nonradiogenic noble gases are overprinted by atmosphere, due to the larger isotopic difference between the mantle and the surface reservoirs.^49^

The fact that all Yellowstone samples, regardless of their Δ_30_ and δ^129^Xe/^130^Xe, fall along a similar trend when comparing their δ^15^N with stable noble gas isotope systematics, indicates that they likely share a similar δ^15^N source composition (Figure 2, 3). Since the composition of the atmospheric component (prior to any fractionation) is known, this suggests that the δ^15^N of the Yellowstone mantle source must be similar to the atmosphere. Previously, mixing relationships in Δ_30_ vs δ^15^N space between a high temperature Yellowstone endmember (with a Δ_30_ of 0‰) and an assumed single fractionated atmospheric endmember, taken as the sample with atmospheric Δ_30_ and the most fractionated (i.e., negative) δ^15^N, defined the δ^15^N of Yellowstone mantle endmember to be ∼ + 3‰ (Figure 2).^24^ However, as we have shown here, there is no single fractionated air component within hydrothermal systems, and instead the degree of fractionation varies across sites and even over time at a single degassing site. It is therefore more justified to take the δ^15^N of the sample with the lowest Δ_30_ to best estimate source nitrogen isotopic compositions.^47^ The Yellowstone sample (Brimstone Basin) with the lowest yet measured Δ_30_ (+3.8‰) has a δ^15^N of +0.2‰,^24^ which is indeed similar to the atmospheric δ^15^N composition of 0‰. However, this sample may contain a crustal nitrogen component, as was previously argued based on its anomalously low ^3^He/^4^He, which could raise the δ^15^N toward heavier values.^57–60^ Considering both the clustering of the low Δ_30_ samples in Figure 2 toward a δ^15^N of approximately – 1‰, and the lack of any significant difference in the trend of high vs low Δ_30_ samples in Figure 3, it is apparent that the δ^15^N of the Yellowstone mantle source must be close to the atmospheric value.^47,56^

If Yellowstone is considered representative of the plume source mantle then a δ^15^N of ∼ 0‰, is slightly lower than the values previously suggested as representative of the plume source mantle (+3.0‰ ± 2.1‰; average and standard deviation from Marty and Dauphas, 2003).^7^ However, if only samples with high ^3^He/^4^He (Loihi and Iceland) similar to Yellowstone are taken (i.e., excluding Ocean Island Basalts with ^3^He/^4^He lower than or equal to MORB, as these could be affected by recycled sedimentary or crustal signatures), then the δ^15^N becomes +0.7‰ ± 1.5‰,^7^ which is within uncertainty of the atmospheric value and similar to the value of the Yellowstone mantle plume estimated in this study. Therefore, there appears to be broad agreement across basalt and hydrothermal gas samples that the primitive plume mantle has a δ^15^N of ∼ 0‰. Interestingly, degassing from a primitive mantle with a δ^15^N of ∼ 0‰, under oxidizing conditions, could potentially account for the composition of the atmosphere, potentially resolving in part the isotope disequilibrium between Earth’s mantle and the surface reservoirs.^61^

### 4.3 The Noble Gas and Nitrogen Elemental Composition of the Plume Mantle Source

Nitrogen to noble gas ratios can provide important constraints on the origin of volatiles in different mantle reservoirs. This is particularly the case for N_2_/^36^Ar since nitrogen and argon have similar solubilities in silicate melts, at least under modern mantle oxygen fugacities, and therefore their relative abundances should not be significantly modified during melting or degassing.^2,62^ Variation in the N_2_/^36^Ar between different mantle reservoirs may represent the preferential addition or removal of either N-bearing species or Ar from the mantle through subduction^7^ or mantle differentiation and core formation.^11^

Using the relationship between Δ_30_ and δ^40^Ar/^36^Ar and δ^129^Xe/^130^Xe (Figure 4) it is possible to constrain the N_2_/^36^Ar and N_2_/^130^Xe ratios of the high temperature, deeply sourced components at Yellowstone. Mixing lines are projected through the data to a hypothetical mantle endmember with a δ^40^Ar/^36^Ar and δ^129^Xe/^130^Xe of ∼ 35000‰ and 76‰ respectively, which represent the isotopic endmember compositions of the Iceland mantle plume source^20^ and is therefore taken as the best estimate for the Yellowstone mantle endmember composition. The curvatures of the mixing lines through the samples represent mixing between an atmospheric and a high temperature component. The curvatures of the mixing lines are defined by (^14^N^14^N/^36^Ar)_HighT_/(^14^N^14^N/^36^Ar)_ASW_ and (^14^N^14^N/^130^Ar)_HighT_/(^14^N^14^N/^130^Ar)_ASW_ for the Figure 4A and 4B, respectively. Calculating the curvature of mixing lines in Δ_30_ vs δ^40^Ar/^36^Ar and δ^129^Xe/^130^Xe space provides a novel way to estimate the N_2_/^36^Ar and N_2_/^130^Xe of the deep reservoirs feeding hydrothermal systems. To compute the N_2_/^36^Ar and N_2_/^130^Xe of the deep endmember we assume that the atmospheric component has a N_2_/^36^Ar and N_2_/^130^Ar composition similar to air saturated water (ASW) at 20 °C and atmospheric ^40^Ar/^36^Ar and ^129^Xe/^130^Xe. We note that the choice of temperature for the ASW component, within the likely range for meteoric fluids in Yellowstone, does not significantly change the N_2_/^36^Ar and N_2_/^130^Ar computed for the mantle endmember. Best-fit mixing hyperbolas were computed by allowing the N_2_/^36^Ar and N_2_/^130^Xe to freely vary, while employing a grid search to find the ratios that minimizes the χ^2^ cost function.^42^

We calculate the N_2_/^36^Ar of the Yellowstone endmember to be 5.3 ± 0.7 × 10^5^ (1σ). This is slightly lower than the previous value calculated by Labidi et al., (2020)^24^ for the Yellowstone mantle source (1.6_–0.7_^+0.4^ x 10^6^). However, the N_2_/^36^Ar estimated previously included samples from Brimstone Basin, which have a large crustal component likely biasing the fit toward higher N_2_/^36^Ar values that are associated with crustal components.^7^ While our fit also contains Brimstone Basin samples, these new samples have N_2_/^36^Ar similar to ASW and therefore do not have a significant influence on the curvature. Our estimate of the Yellowstone mantle source N_2_/^36^Ar is similar to previous determinations for plume influenced basaltic samples (∼3 × 10^5^)^48^ and lower than the value for the convecting MORB mantle (2.0_–1.2_^+1.0^ x 10^6^)^24^ or the Eifel mantle source in Germany (4.7_–1.6_^+0.8^ x 10^6^).^24^ We note that increasing or decreasing the ^40^Ar/^36^Ar value assigned to the mantle endmember will result in a correlated increase or decrease in the calculated N_2_/^36^Ar. However, since the δ^40^Ar/^36^Ar of the Iceland mantle endmember is similar or greater than that of other mantle plume endmembers including: the Kola Plume (∼+15000‰),^63^ Réunion (∼+35000‰),^64^ and Galapagos (+19000 – + 26000‰),^65^ changing the mantle endmember ^40^Ar/^36^Ar value used in the calculations to that of another plume would only serve to lower the calculated N_2_/^36^Ar, and further distinguish it from the convecting upper mantle.

From the curvature of the mixing line in Figure 4B, we estimate the N_2_/^130^Xe of the Yellowstone mantle source to be 8.5 ± 1.1 × 10^8^. This overlaps within uncertainty to the N_2_/^130^Xe previously calculated for the bulk ((1.9 ± 1.0) × 10^9^) and the depleted ((1.1 ± 1.0) × 10^9^) mantle.^10^ Similar to the situation for ^40^Ar/^36^Ar, changes to the ^129^Xe/^130^Xe mantle endmember composition used in these mixing calculations would change the curvature and modify the calculated N_2_/^130^Xe of the Yellowstone mantle source. However, once again the Iceland ^129^Xe/^130^Xe endmember value used in the mixing calculation is greater than that determined for other plume mantle sources, including Réunion (∼68‰)^64^ and Galapagos (32‰).^66^ The N_2_/^130^Xe calculated here is therefore likely a maximum, although using the lower ^129^Xe/^130^Xe endmember value of Réunion instead of Iceland for example would not significantly change the calculated N_2_/^130^Xe. The general similarity in these two estimations (this study and Marty, 2012),^10^ which use completely different methods, suggests there is a growing confidence in our knowledge of the nitrogen to noble gas ratio of the mantle. Finally, using calculated endmember N_2_/^36^Ar and N_2_/^130^Xe ratios, it is possible to compute the ^36^Ar/^130^Xe of the Yellowstone mantle source to be 1611 ± 212, which is also within the range of previous plume estimates (1576 ± 1 for Galapagos and 1514 ± 1 for Iceland).^66^ This is distinct from that measured in CO_2_ well gases (957 ± 82)^22^ and MORB popping rock (970 measured in the step crush release with the highest ^20^Ne/^22^Ne),^67^ once again indicating that Yellowstone originates from a distinct plume mantle reservoir similar to Galapagos and Iceland.

### 4.4 Reconciling Heterogeneous Nitrogen and Noble Gas Compositions in the Mantle

The primitive plume mantle appears distinct from the convecting MORB mantle in terms of its nitrogen isotopic composition (∼0‰ for the plumes source vs – 5‰ in the MORB source),^7,14–17,48,68^ as well as its N_2_/^36^Ar and ^36^Ar/^130^Xe compositions. The difference in δ^15^N between the MORB and plume mantle source has historically been attributed to the preferential addition of isotopically heavy nitrogen from oceanic sediments (δ^15^N = +3 to +7 ‰)^69,70^ into the deep mantle.^7,8,71^ If the deep mantle originated with an isotopically lighter δ^15^N signature, perhaps similar to a primordial component found in enstatite chondrites^72^ that was suggested to be present in some very rare diamond samples (−40‰ to ∼ – 25‰),^73^ then the preferential addition of isotopically heavy nitrogen to the deep mantle could have progressively raised the δ^15^N of the deep mantle. However, the broad similarity in N_2_/^3^He between plume and MORB mantle, as well as the lack of variation in δ^15^N across basalt samples with variable K_2_O/TiO_2_, which traces the addition of recycled material to a mantle source, appear inconsistent with the preferential addition of isotopically heavy, nitrogen-rich sediments to the mantle plume source.^17,24,74^ Furthermore, as we have shown here, the plume mantle source appears to have elevated ^36^Ar/^130^Xe compared to the MORB mantle source. Since Xe is likely to be more efficiently recycled to the mantle through subduction than Ar,^75^ the addition of Xe-enriched recycled material to the plume mantle is also unlikely to explain the difference in ^36^Ar/^130^Xe between the plume and MORB mantle sources.^76^

Another hypothesis that could explain the dichotomy between plume and MORB mantle source compositions with respect to nitrogen and noble gases is the preferential sequestration of nitrogen into the core.^10,77^ This has been suggested to account for the depletion of nitrogen in the Bulk Silicate Earth (BSE) relative to chondritic abundance patterns of carbon and noble gases.^10^ Nitrogen isotope fractionation between metal and silicate materials could drive the mantle composition toward heavier values.^11–13^ Therefore, the heavier δ^15^N in the primordial plume mantle, when compared to MORB, could be the result of greater nitrogen partitioning into metal phases in the lower mantle during core formation. The extent to which N isotopes fractionate during core-mantle differentiation is debated but it appears that the δ^15^N of the silicate mantle should be enriched by +1.0 to +5.5 ‰ relative to the Fe-rich metal core.^11,13^ The N isotopic composition of the mantle therefore broadly reflects the composition of the accretionary building blocks that made the Earth,^13^ although an enhanced role in metal-silicate partitioning and core formation in the deep plume mantle could potentially explain the difference in δ^15^N between the MORB and plume mantle reservoirs.

Core formation has also been proposed to explain the depletion of Xe on Earth when compared to other noble gases, the so-called “missing Xe problem”.^78^ The elevated ^36^Ar/^130^Xe in the Yellowstone mantle source, as well as other mantle plumes, when compared to MORB^76^ could also be the result of enhanced partitioning of Xe into the metal phases in the lower primordial mantle. If the plume mantle source experienced a greater degree of metal-silicate partitioning during core formation than the convecting MORB mantle then this could explain both the enriched δ^15^N and elevated ^36^Ar/^130^Xe of the plume mantle source when compared to the MORB mantle. While Xe could be incorporated into metal phases in the mantle during core formation it remains unclear whether this process could also fractionate noble gas elemental ratios^79^ sufficiently to explain the ∼ 10-fold depletion of Xe relative to the other noble gases on Bulk Silicate Earth.^80^ The role of core formation in setting volatile heterogeneities in the mantle therefore remains unclear.

Finally, if N isotopes in the mantle primarily reflect the composition of accretionary building blocks, one can consider whether the heterogeneous nitrogen to noble gas ratios in the mantle also reflect primordial accretionary compositions.^24^ The N_2_/^36^Ar of Yellowstone mantle source (5.3 ± 0.7 × 10^5^) is indistinguishable from the range of chondritic values, which is true also for the MORB mantle.^24,81^ Since N_2_ and Ar have similar solubilities in silicate melts under oxidative mantle fugacities,^2,62^ the inherited chondritic N_2_/^36^Ar of the mantle may have been preserved throughout Earth’s history, as long as magmatic degassing has occurred under near constant mantle oxygen fugacities.^82^ Subtle difference in the N_2_/^36^Ar and ^36^Ar/^130^Xe between the plume and MORB mantle domains could therefore be explained by preferential loss of N_2_ and Xe from the plume source during core formation or the preferential addition of recycled material enriched in N_2_ and Xe to the upper MORB mantle during subduction. Given the similarity in N_2_/^36^Ar between both mantle reservoirs and chondrites, we propose that the nitrogen isotopic composition of the mantle may broadly reflect the primordial accretionary composition of Earth. The δ^15^N of the mantle, lying somewhere between – 5‰ and 0‰, would therefore preclude the requirement of enstatite chondrite derived nitrogen (average δ^15^N of ∼ – 20‰) as a building block for N within Earth. Nitrogen on Earth may therefore require the addition of ^15^N-rich material potentially in the form of carbonaceous chondrite-like material from the outer solar system.^83,84^

## 5 Conclusion

By coupling high precision noble gas analyses with nitrogen isotopologues, we have provided new insights into the physical processes occurring within hydrothermal systems at Yellowstone. We demonstrate that nitrogen isotopes in hydrothermal gas samples from Yellowstone are isotopically fractionated by diffusional transport fractionation (DTF) processes (through rising magmatic CO_2_), which can account for much of the variation in δ^15^N measured in hydrothermal gas samples.

Using the strong correlation between Δ_30_ and δ^129^Xe/^130^Xe in Yellowstone gases, we show that Δ_30_ is an effective tracer of mantle nitrogen. Despite the presence of mantle nitrogen in some of Yellowstone samples, all samples, including those with atmospheric Δ_30_ and δ^129^Xe/^130^Xe, fall on a similar fractionation line when δ^15^N is plotted against the stable noble gases (Figure 3). This demonstrates that the Yellowstone mantle source has a δ^15^N similar to the atmosphere (i.e., ∼ 0‰).

From the curvature of mixing lines when Δ_30_ is plotted against δ^40^Ar/^36^Ar and δ^129^Xe/^130^Xe (Figure 4), we show that the N_2_/^36^Ar, N_2_/^130^Xe and ^36^Ar/^130^Xe are similar to previous estimates derived for the plume mantle source from rock and gas analyses, thereby confirming that hydrothermal gases from Yellowstone are sourced from the deep primitive plume source mantle. This study demonstrates that coupling of high precision noble gas analyses with nitrogen isotopologues in hydrothermal gas samples can help identify mantle source signatures even in samples with large degrees of atmospheric contamination.

We demonstrate that the N_2_/^36^Ar (5.3 ± 0.7 × 10^5^) ^36^Ar/^130^Xe (1611 ± 212) of the Yellowstone mantle source is lower and greater than the MORB mantle source respectively, indicating that the plume mantle source is unlikely to have been more efficiently overprinted from the addition of N_2_ and Xe-rich recycled material. Conversely, we suggest that the δ^15^N and N_2_/^36^Ar of the mantle primarily reflects the composition of the accretionary building blocks that formed Earth, perhaps requiring a flux of carbonaceous chondrite-like material from the outer solar system.

## Supplementary Material





## Notes

Additional figures, showing Kr isotope measured in hydrothermal gas samples from Yellowstone National Park (S1) and stable Ar and Kr isotope variations in hydrothermal gas samples from across the globe measured by dynamic mass spectrometry (S2) (PDF)
All data presented in this study are reported within the supporting excel file (XLSX)

## Notes

For Submission to Special Issue in Honor of Reika Yokochi in ACS Earth and Space Chemistry.
