# Supplementary material for: Noble Gas Isotopes and Nitrogen Isotopologues Reveal Deep Sources and Subsurface Fractionation in Yellowstone Gases
Source: ACS Earth Space Chem. 2025 Mar 24;9(6):1310–21. doi: 10.1021/acsearthspacechem.4c00349 (PMC12183726; doi:10.1021/acsearthspacechem.4c00349)
Supplement: Supplementary file 1 [file sp4c00349_si_001.pdf]

Noble gas isotopes and nitrogen isotopologues reveal deep sources and subsurface fractionation in Yellowstone gases

Michael W. Broadley[1,2*], Peter H. Barry[2], Rebecca L. Tyne[1,2], David V. Bekaert[3], Ruta Karolyte[4], Michael R. Hudak[5], Katelyn McPaul[2], Carlos J. Ramirez[6], J. Curtice[2], Karen G. Lloyd[7], Christopher J. Ballentine[4], Bernard Marty[3], Edward D. Young[8] & Alan M. Seltzer[2]

[1] Department of Earth and Environmental Science, University of Manchester, UK, M13 9PL
[2] Marine Chemistry and Geochemistry, Woods Hole Oceanographic Institution, USA, 02543
[3] Universite de Lorraine, CNRS, CRPG, Nancy, France, 54500
[4] Department of Earth Science, University of Oxford, UK, OX1 3AN
[5] Geosciences Department, Williams College, USA, 01267
[6] Servicio Geológico Ambiental (SeGeoAm) Heredia, Costa Rica, 40101
[7] University of Southern California, Los Angeles CA, USA, 90007
[8] Earth, Planetary and Space Science, UCLA, USA, 90095

For Submission to Special Issue in Honor of Reika Yokochi in ACS Earth and Space Chemistry

*michael.broadley@manchester.ac.uk

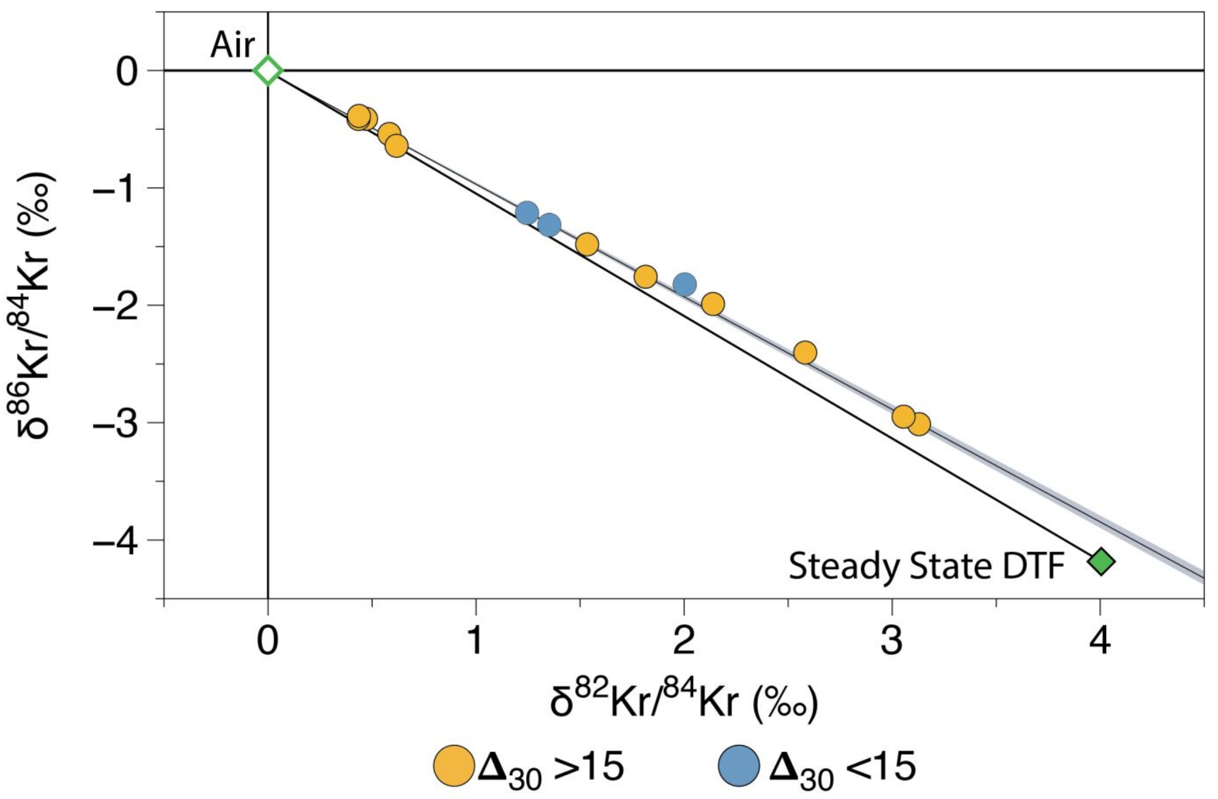

Figure S1. Kr isotope (in per mil deviations from atmosphere) measured in hydrothermal gas samples from Yellowstone. The data fall very close to the expected DTF fractionation line and never exceed the steady state DTF value. Isotopic fractionation of noble gases in hydrothermal gas is therefore likely controlled by DTF even if the ease with which a specific element reaches steady state DTF is variable. Linear regression represents an error weighted fit shown with 1σ uncertainty envelope. Uncertainties for the samples are reported to 1σ and are often smaller than symbol size.

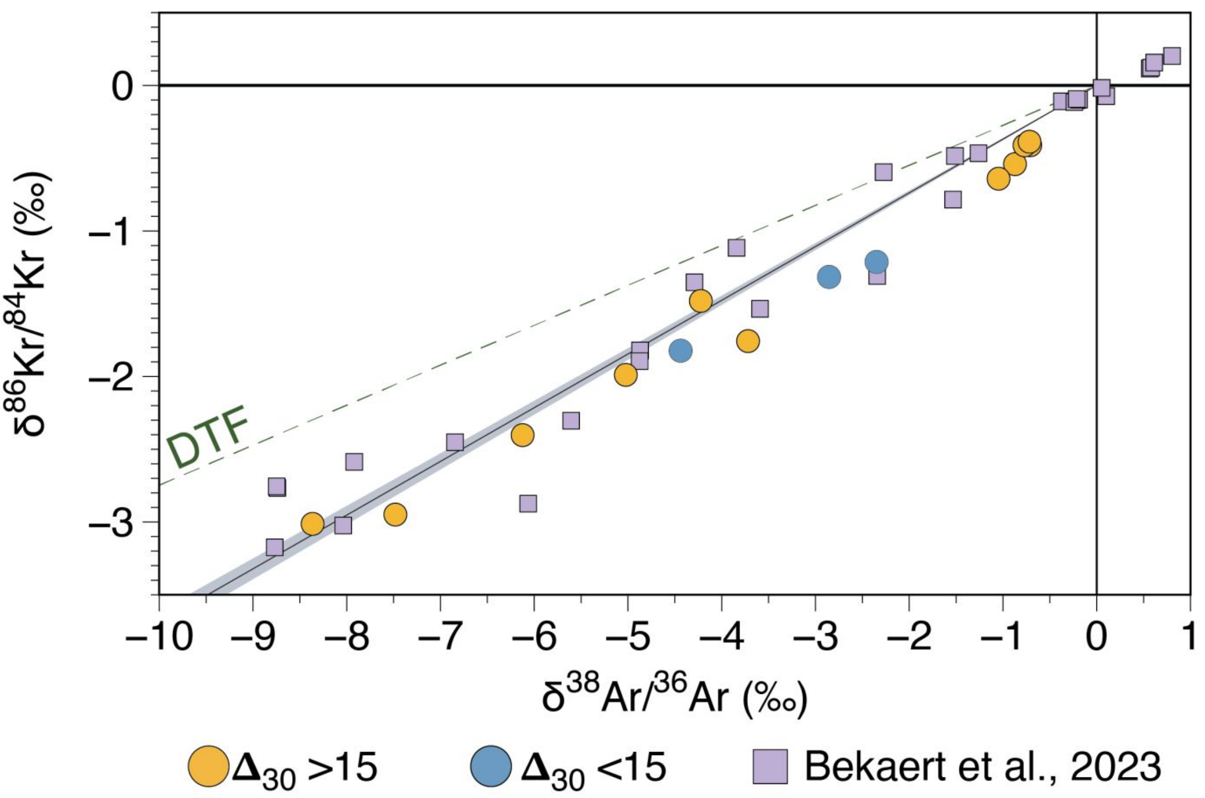

Figure S2. Stable Ar and Kr isotope variations measured in hydrothermal gas samples from across the globe. The data presented represent all the published data for hydrothermal gas samples analysed by ultra-high precision dynamic mass spectrometry[1]. Linear regression represents an error weighted fit shown with 1σ uncertainty envelope. Uncertainties for the samples are reported to 1σ and are often smaller than symbol size.

<u>References</u>

1. Bekaert, D.V., Barry, P.H., Broadley, M.W., Byrne, D.J., Marty, B., Ramírez, C.J., de Moor, J.M., Rodriguez, A., Hudak, M.R., Subhas, A.V. and Halldórsson, S.A., 2023. Ultrahigh-precision noble gas isotope analyses reveal pervasive subsurface fractionation in hydrothermal systems. Science Advances, 9(15), p.eadg2566.
